# The Non-Survival Effects of Glial Cell Line-Derived Neurotrophic Factor on Neural Cells

**DOI:** 10.3389/fnmol.2017.00258

**Published:** 2017-08-22

**Authors:** Daniel Cortés, Oscar A. Carballo-Molina, María José Castellanos-Montiel, Iván Velasco

**Affiliations:** ^1^Instituto de Fisiología Celular—Neurociencias, Universidad Nacional Autónoma de México México City, Mexico; ^2^Laboratorio de Reprogramación Celular del IFC-UNAM, Instituto Nacional de Neurología y Neurología México City, Mexico

**Keywords:** neurogenesis, electrophysiological maturation, motor neuron, dopaminergic neurons, enteric nervous system, pain, neuroblastoma, glioblastoma

## Abstract

Glial cell line-derived neurotrophic factor (GDNF) was first characterized as a survival-promoting molecule for dopaminergic neurons (DANs). Afterwards, other cells were also discovered to respond to GDNF not only as a survival factor but also as a protein supporting other cellular functions, such as proliferation, differentiation, maturation, neurite outgrowth and other phenomena that have been less studied than survival and are now more extendedly described here in this review article. During development, GDNF favors the commitment of neural precursors towards dopaminergic, motor, enteric and adrenal neurons; in addition, it enhances the axonal growth of some of these neurons. GDNF also induces the acquisition of a dopaminergic phenotype by increasing the expression of Tyrosine Hydroxylase (TH), Nurr1 and other proteins that confer this identity and promote further dendritic and electrical maturation. In motor neurons (MNs), GDNF not only promotes proliferation and maturation but also participates in regenerating damaged axons and modulates the neuromuscular junction (NMJ) at both presynaptic and postsynaptic levels. Moreover, GDNF modulates the rate of neuroblastoma (NB) and glioblastoma cancer cell proliferation. Additionally, the presence or absence of GDNF has been correlated with conditions such as depression, pain, muscular soreness, etc. Although, the precise role of GDNF is unknown, it extends beyond a survival effect. The understanding of the complete range of properties of this trophic molecule will allow us to investigate its broad mechanisms of action to accelerate and/or improve therapies for the aforementioned pathological conditions.

## Introduction

Glial cell line-derived neurotrophic factor (GDNF) is a protein that was initially characterized as a soluble factor extracted from the conditioned media of striatal astrocytes, and once purified, it promoted the survival of dopaminergic neurons (DANs) in culture (Lin et al., 1993).

Afterwards, DANs are not the only responsive cells, but many other cell types are targeted by this trophic factor, even cells located outside the central nervous system (CNS). For example, a lack of GDNF produces renal agenesia (Moore et al., 1996; Pichel et al., 1996; Sanchez et al., 1996). In the nervous system, other cells that respond to GDNF include cerebellar (Tolbert et al., 2001; Subramaniam et al., 2008), hippocampal (Martin et al., 1995; Miyazaki et al., 1999), sensory (Trupp et al., 1995) cortical (Konishi et al., 2014), spinal motor (Henderson et al., 1994) and enteric (Moore et al., 1996) neurons.

GDNF canonically signals via GFRα1 that in turns activates the co-receptor RET. The receptor and the co-receptor are not required to be expressed in the same cell (*cis*), and they also transduce signals in *trans* (i.e., Ret is expressed by one cell and GFRα1 is expressed in a nearby cell; Paratcha and Ledda, 2008). The activation of this receptor complex is associated with downstream pathways, importantly MAP kinases and Akt. This signaling cascade has been reviewed extensively elsewhere (Wang, 2013; Ibáñez and Andressoo, 2017).

The mechanisms underlying the survival-promoting actions of GDNF have been widely studied, but other effects triggered by this factor remain unidentified (Li et al., 2014; Ibáñez and Andressoo, 2017). For example, the expression patterns of the mRNAs (Springer et al., 1994; Choi-Lundberg and Bohn, 1995) and proteins (Kawamoto et al., 2000; Saavedra et al., 2008) for GDNF and its receptors (Quartu et al., 2007), suggest roles in addition to its survival effects. In fact, new functions associated with changes in the expression of downstream effector genes were recently described, such as promotion of proliferation and specification, neurite growth, synaptic and electrophysiological maturation, soma expansion and expression of phenotype-specific proteins. Additionally, GDNF might participate in both homeostasis (pain modulation (Merighi, 2016)) and pathophysiological states (depression (Sharma et al., 2016) and attention-deficit/hyperactivity disorder (Bilgiç et al., 2017)). Because most of the GDNF-associated research has been centered on the promotion of neuronal survival, with a substantial body of knowledge about this function, this review does not cover neuroprotection, but we aim to review the non-survival effects of GDNF.

## Effects of GDNF on Different Scenarios

**Figure 1:**
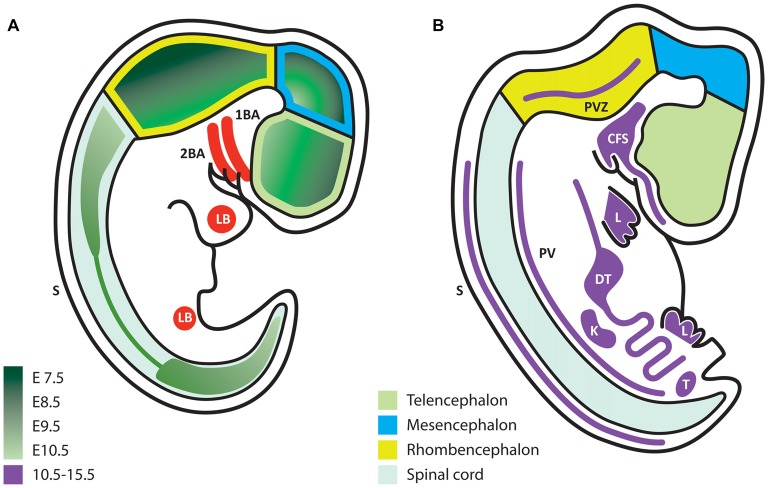
Glial cell line-derived neurotrophic factor (GDNF) expression during embryonic development. **(A)** Schematic representation of embryonic stages E7.5–10.5. GDNF expression appears first in the ventral forebrain and then extends caudally and dorsally according to the color code in green, which indicates the first time of appearance for each region. **(B)** Scheme that represents the expression of GDNF (purple) at later stages (E10.5–15.5). In contrast to early stages, at this time expression is mostly extra-neural. 1BA, first branchial arch; 2BA, second branchial arch; LB, limb bud; S, dorsolateral portion of somites; PVZ, periventricular zone; CFS, craniofacial structures; L, limb; DT, digestive tube; PV, paravertebral mesenchyme; K, kidney; T, testis.

During development, the expression of GDNF and its receptors suggest that they exert non-survival functions, according to the place and stage of expression. Some authors have studied early stages of neural development at embryonic day (E) 7.5–E10.5 and later periods at E10.5–E15.5, when postmitotic neurons appear (Hellmich et al., 1996). During the formation of the neural plate, primary and secondary neurulation end at E9.5 in the mouse (Greene and Copp, 2014). During this same period, neural precursors are still proliferating and start to acquire regional commitment; however, at this stage, no postmitotic neurons are observed, and therefore axonal growth does not occur. Thus, the neuronal survival effect associated with trophic factors such as NGF, GDNF and NT-3, among others, is not present at this point. Any factor expressed at this time should serve other functions rather than neuronal survival. Using *in situ* hybridization, the *Gdnf* mRNA is detected beginning at E7.5 (the earliest stage analyzed) and reaches the highest level at E9.5 (Figure 1A), before decreasing at E10.5 (Figure 1B). In these early stages, the *Gdnf* mRNA is specifically expressed within the developing neural tube: it begins in the forebrain, and later extends to the midbrain and finally throughout the neural tube, with higher expression in dorsal regions (Figure 1A).

Although this review does not presume to cover the extra-neural functions of GDNF we must mention that from E10.5 to E15.5, GDNF is predominantly expressed in extra-neural regions, with the exception of two small regions in the hindbrain (Figure 1B). GDNF expression in peripheral tissues might influence their innervation at later stages. For example, mesenchymal tissues (such as limb buds) will be directly contacted by axons, which respond to GDNF during navigation. At later stages, muscles express alternating patterns of GDNF in the dorsal or ventral limbs at a time when motor neurons (MNs) send axons to reach their muscular targets.

When postmitotic neurons start to appear, organogenesis is exhibiting its maximum activity. In the kidney, GDNF expression is associated with epithelial-mesenchymal interactions, and the absence of this gene causes renal agenesia (Sariola and Saarma, 2003). On the other hand, the gut expresses GDNF from E9.5 to E11.5. In this tissue, GDNF promotes the formation of an extensive autonomously functioning nervous plexus; in the absence of GDNF signaling pathways, this plexus fails to develop, leading to Hirschsprung disease (Martucciello et al., 2000). Many other organs with less innervation that also require motor visceral, sympathetic or parasympathetic innervation must attract innervating axons. Studies examining the tissues in which GDNF or other trophic factors participate in innervation would be interesting. Trupp et al. (1995) have reported patterns of GDNF expression in target organs for sensory neurons (SN) and were the first to suggest that GDNF contributes to the attraction of nascent axons. Although GDNF Knock-Out (KO) studies have been reported, innervation has not been studied sufficiently in other organs that express GDNF, with the exception of the major alterations observed in the cervical sympathetic ganglia (Moore et al., 1996) or the sympathetic trigeminus (Treanor et al., 1996). The effects of a GDNF deletion might not be noticeable because of redundant signals or because target organs function with reduced innervation.

The innervation of several tissues is associated with chemotropic molecules. Activation of GDNF signaling in *trans* has been proposed to participate in promoting the connectivity between different regions of the brain, as well as with extra-neural targets. Examples of activation in *trans* have been observed in vibrissae innervated by the trigeminal nerve, in the lateral septum innervated by the CA3 region of the hippocampus, and the fourth layer of the cerebral cortex receiving axons from the thalamus (Yu et al., 1998). Thus, GDNF might be acting as a chemotropic molecule. Notably, Ikeda attested that GDNF is expressed in the corpus callosum at early postnatal periods of high axon branching, but in this case, the exact role of this trophic factor is unknown (Ikeda et al., 1999).

Some functions of downstream genes related to axonal growth and guidance have been identified following the activation of GDNF signaling and analysis of gene expression. GDNF overexpression in a neural precursor cell (NPC) line regulates 283 genes (43 specific to GDNF compared to CNTF). GDNF down-regulates genes associated with cortical layer development, cytoskeletal reorganization and axonal stabilization, but up-regulates proteins related to the extracellular space and cell surface, axonal sprouting, neurite outgrowth and spine formation, which might contribute to the ability of axons to sense the extracellular environment and continue growing (Pahnke et al., 2004). Functional studies are ongoing to corroborate these effects on axonal guidance and migration for potential use in regenerative medicine (Marquardt et al., 2015; Qin et al., 2016; Santos et al., 2016) and treatments for nerve and/or spinal cord injury; several reviews on this matter are available (Gordon, 2009; Forostyak et al., 2013; Awad et al., 2015).

In addition to the effects of GDNF on CNS development, studies showing transdifferentiation have recently been reported. These methods avoid ethical issues (human embryonic stem cells (ESCs)) or the requirement for reprogramming to a pluripotent state (induced Pluripotent Stem Cells) to produce human neurons. In this regard, the acquisition of a neuronal phenotype has been achieved from fibroblasts using transcription factors (Vierbuchen et al., 2010) or small molecules (Li et al., 2015). Trophic factors appear to be dispensable for direct conversion to neurons. However, Zhu T. et al. (2015) transdifferentiated mesenchymal stem cells to neurons by overexpressing GDNF and NT-3 in a fetal gut culture medium. Neuronal markers and Na and K currents were detected. Thus, GDNF and NT-3 modulate gene expression and the epigenetic landscape, but the underlying mechanism must still be examined.

## Dopaminergic System

**Figure 2:**
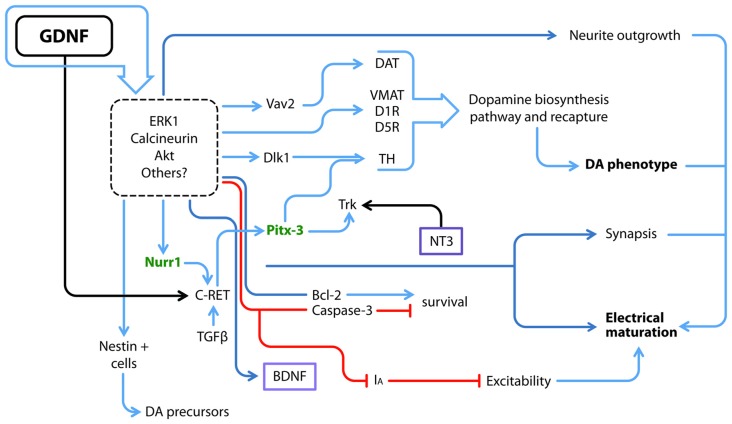
GDNF and its downstream molecular interactions. GDNF regulates several proteins and transcription factors via ERK, Calcineurin, AKT and probably other pathways; some of GDNF actions have been directly related to at least one of these transducing pathways, but not all of them have been elucidated (dotted square). Utterly what GDNF promotes is the electrical and synaptic maturation, as well as survival and the acquisition of a dopamine (DA) neuron phenotype. Blue arrows denote a positive interaction, red lines depict a negative effect and black arrows a positive interaction by binding of each ligand to its receptor. Purple boxes denote soluble factors. Green letters denote transcriptional factors that maintain the DA phenotype throughout the life of animals. DAT, DA transporter; D1R, DA receptor type 1; D5R, DA receptor type 5; *I*_A_, A-type potassium channel; TH, Tyrosine Hydroxylase; VMAT, Vesicular monoamine transporter.

The initial trophic characterization of GDNF was performed in midbrain dopaminergic neuronal cultures; in this seminal work, GDNF not only promoted DANs survival but also soma enlargement and neurite outgrowth (Lin et al., 1993). Since then, new GDNF actions such as proliferation, maturation and long-term effects have been studied in different model systems, as summarized in Figure 2.

The development of midbrain DANs is a process that occurs in the embryo and therefore the isolated effects of different added factors are difficult to test. Several studies have isolated the midbrain to obtain neural precursors or DANs in an attempt to circumvent this problem. These studies have described pro-survival and/or pro-differentiation actions on embryonic cells of BDNF, TGFα, bFGF, NT-3, NT-4/5, EGF, PDGF, IGF and IL-6, all together or in several combinations with limited numbers of trophic factors (Hyman et al., 1994; Engele and Schilling, 1996; Engele, 1998). However, which of these proteins are pro-survival factors and which are pro-differentiation factors? Are they redundant signals? The answers have not been completely revealed. BDNF and NT-3 do not exert additive effects on the maturation of DANs (Hyman et al., 1994). Moreover, BDNF, NT-3, bFGF and GDNF selectively stimulate different DAN subpopulations (Engele and Schilling, 1996).

The previous data describe effects on postmitotic cells, whereas other authors have cultured neural stem/precursor cells obtained from the ventral midbrain, the area in which mesencephalic DANs are born, in the presence of GDNF. This stimulation promotes the expansion of dividing nestin-positive neural precursors and increases the neurosphere diameter; however, this latter increase may also be associated with an anti-apoptotic response, since Bcl-2 expression is up-regulated and caspase-3 expression is down-regulated (Figure 2; Lei et al., 2011). Ling et al. (1998) have described culture conditions for DANs that yield <5% of cells that express the rate-limiting enzyme in dopamine (DA) biosynthesis, Tyrosine Hydroxylase (TH). The addition of medium conditioned with mononuclear cells induces an increase in TH immunoreactive cells, an effect probably caused by LIF, IL-1 and IL-11, since each protein by itself increased the number of TH+ cells. Nonetheless, the TH+ cells produced by stimulation with LIF, IL-1 and IL-11 exhibit an immature morphology. When GDNF is added together with LIF and these ILs, more neurites emerge in the culture (Ling et al., 1998). However, the use of several cytokines and trophic factors together as described above makes the dissection of their individual molecular effects difficult.

As shown in the study by Roussa and Krieglstein (2004), GDNF does not increase TH expression in neurospheres derived from the ventral midbrain, but increases the expression of Nurr1 and Pitx3, the latter of which is a transcriptional factor that is part of the regulatory complex that maintains the dopaminergic phenotype. More recently, Nurr1 was reported to induce the expression of the co-receptor for GDNF, c-Ret. Nurr1-expressing cells exposed to GDNF are eventually more responsive to GDNF, contributing to the up-regulation of Pitx3 expression (Peng et al., 2011) and subsequently consolidating the dopaminergic phenotype. In parallel, GDNF also increases DA transporter (DAT) activity (Zhu S. Y. et al., 2015; Figure 2). Differences might arise because the studies were performed at different stages, since DA-synthetizing enzymes are rarely detected at E12–E14 compared to later stages (Schaller et al., 2005). Microarrays have corroborated that GDNF signaling favors the expression of genes associated with DA biosynthesis and recapture, as well as calcineurin. Inhibitors of ERK1/2 prevented these effects (Consales et al., 2007). Thus, GDNF participates in proliferation and the acquisition of a dopaminergic phenotype. The mechanism by which GDNF interacts with other factors to promote this phenotype is still a matter of study.

A GDNF-expressing virus injected in the mouse adult striatum exhibits increased expression in both the striatum and *substantia nigra*. Concomitantly, increases in c-Ret, GFRα1 and TH expression were reported. Notably, Delta-like 1 (Dlk1) homolog showed a higher up-regulation. Dlk1 is expressed in the developing mouse ventral midbrain in close apposition with DANs. Treatment of a human mesencephalic cell line with GDNF significantly increased dopaminergic differentiation and Dlk1 expression; practically all TH+ neurons were positive for Dlk1 as well (Christophersen et al., 2007; Figure 2). Thus, GDNF might direct the commitment of neurons towards specific phenotypes in addition to increasing neuronal survival.

In neurons, the outgrowth of the dendritic tree occurs in parallel with electrophysiological maturation, which in turn facilitates DA release after potassium depolarization. A GDNF or BDNF treatment significantly increases DA release from ventral mesencephalic cultures (Feng et al., 1999); GDNF enhances the quantal size of DA release in postnatal cultures (Pothos et al., 1998). Consistent with these findings, an injection of GDNF in the *susbtantia nigra* of adult rats increases DA release *in vivo* and promotes behavioral consequences such as an increase in spontaneous locomotor activity and higher velocity of movement (Hebert et al., 1996). The effects of GDNF were tested on dopaminergic and GABAergic neurons derived from the ventral tegmental area and cultured in microislands; these neurons co-release glutamate and establish synapses on themselves. By measuring autaptic currents, GDNF increases the amplitude of excitatory currents and the frequency of miniature currents. The aforementioned features are driven by GDNF and only observed in DANs, but not in GABAergic neurons (Bourque and Trudeau, 2000). GDNF also modulates excitability in an acute fashion by inhibiting A-type K^+^ channels, an ERK-mediated effect (Yang et al., 2001). Experiments such as those described above show that GDNF modifies the synaptic anatomy and favors electrophysiological maturation.

ESCs differentiate into tissues derived from the three embryonic layers under specific conditions, and they are capable of producing a fertile adult organism in chimeric studies. These cells have been used to study differentiation processes *in vitro* because they emulate the events that occur during normal development. However, the use of several growth factors and the presence of serum in the differentiation protocols hamper a clear interpretation of the individual roles of each factor in cell commitment or survival. For example, according to the study by Rolletschek et al. (2001), a cocktail including GDNF promotes the expression of the pro-dopaminergic gene Nurr1 in neural precursors. Nurr1 is expressed from E10 in the ventral midbrain, prior to the appearance of TH+ cells, and activates the TH promoter. Consistent with these findings, an increase in TH expression was reported; in parallel, Bcl-2 expression was also up-regulated at terminal differentiation stages. In this case, the survival-promoting factors are also involved in differentiation. Other studies have implicated GDNF in dopaminergic induction.

The addition of GDNF to cultures of mouse ESCs grown on stromal PA6 cells (a well-known cell line able to induce the neuronal differentiation of pluripotent cells) increases TH immunoreactivity (Buytaert-Hoefen et al., 2004). Similar to dopaminergic induction by PA6 cells, co-culture of cynomolgus monkey-derived ESCs on Sertoli cells yields a high number of DANs (60% TH+/TUJ1+) in only 3 weeks, with no further additions of other factors. Sertoli cells secrete GDNF, and GDNF-neutralizing antibodies block dopaminergic differentiation by 50% (Yue et al., 2006), indicating that GDNF contributes to this induction, but other factors, such as physical contact, also contribute. NPCs engineered to secrete GDNF promote the differentiation of ESC-derived NPCs to DANs in a co-culture system (Ostenfeld et al., 2002). The addition of recombinant GDNF to differentiating human NPCs derived from ESCs promotes a higher level of dopaminergic differentiation after 21 days in culture. GDNF increases the expression of En-1, Nurr1, Pitx3, Vmat and TH (Young et al., 2010; Figure 2).

## Motor Neurons and The Spinal Cord

**Figure 3:**
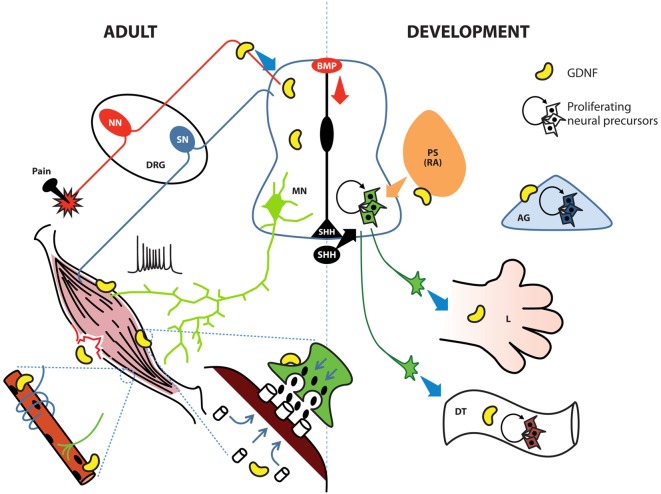
GDNF possesses diverse roles in the adult spinal cord, the developing neural tube and their associated tissues. During development (right side), the notochord and floor plate secrete sonic hedgehog (SHH) to induce ventralization of the neural tube; retinoic acid (RA) induces caudalization. During early stages, GDNF induces the proliferation of neural precursors to increase the pool of these cells in the ventral neural tube, digestive tube (DT) and adrenal gland (AG). In later stages GDNF contributes to protracting motor neuron (MN) axons to limb (L) bud, specific group of muscles, or the enteric nervous system (ENS). In adult organisms (left side), GDNF is produced by Clarke cells in the central spinal cord and also by interneurons; in the neuromuscular junction (green terminal with post-synaptic muscle in brown), GDNF facilitates the release of Acetylcholine (ACh) by MNs. In muscle, GDNF promotes the clustering of ACh receptors, the innervation of sensory neurons (SN), and the connection between alpha MNs and intrafusal muscle fibers (blue ribbon surrounding the muscle innervated by a green MN). GDNF also contributes to muscular recovery after delayed-onset of muscular soreness or injury. Finally, GDNF is secreted by peptidergic neurons in the dorsal root ganglia (DRG) to modulate pain threshold. BMP, bone morphogenetic protein; NN, nociceptive neurons; PS, paraxial somite.

MNs are a diverse group of cells distributed throughout many regions of the central and peripheral nervous system, controlling all motor behaviors throughout the organism. GDNF exerts its effects before MNs undergo terminal differentiation. As shown in our recent study, GDNF overexpression in ESCs increases the proliferation of MN-committed neural precursors (Olig2+; Cortés et al., 2016). Olig2 maintains the cycling of MN precursors (pMNs) and precludes the expression of both pro-neural factors such as neurogenin 2, and HB9, a pro-MN factor (Lee et al., 2005). Hence, by increasing Olig2 expression, GDNF promotes the replication of pMNs (Figure 3). Moreover, transient exposure of pluripotent ESCs to GDNF yielded a higher number of MNs (Cortés et al., 2016), strongly suggesting that GDNF primes these undifferentiated cells to differentiate into the neural lineage. GDNF and its receptors are expressed in early stages of development of the ventral neural tube, when pMNs are leaving the cell cycle (Ortega-de San Luis and Pascual, 2016). According to Shimada, GFRα1 expression in the ventral neural tube is independently up-regulated by Ascl1, NeuroM and Ngn2. The expression of each of these factors is mutually exclusive, since each one marks a temporary window in development; Ascl1 is the first factor to be expressed when pMNs are proliferating (Shimada et al., 2012) Thus, GDNF seems to act before the terminal differentiation of MNs, supporting pMN cycling during development when Ascl1 and Olig2 are active to the point when Ngn2 and Hb9 ceases proliferation of pMNs. At this point, young postmitotic neurons must acquire electrical maturity. As shown in our previous study, constitutive GDNF secretion promotes the maturation of ESC-derived MNs. The observed properties we reported include increased evoked and spontaneous action potentials, resistance to spike inactivation and electrical signatures of MNs, such as I-H current, rebound action potential and spike frequency adaptation (Cortés et al., 2016). Hence, GDNF is acting at different stages of MN development; its function in each specific stage should be determined by different intracellular responses and/or the expression of specific transcription factors unique to every time point during development.

During development, the expression patterns of GDNF and its receptors undergo time- and tissue-dependent changes (Kanning et al., 2010). Hox genes pattern the anteroposterior axis of the body during early development, but this pattern also directs the arrangement of the MN subpopulation and its muscular targets by controlling RET/GFRα1 expression; mutations in Hox genes also affect neuromuscular configurations (Catela et al., 2016). Regarding GDNF, the earliest extra-neural source of this protein for limb MNs is detected at E10 in the limb bud; E10 is the time when axons begin to outgrow (Kramer et al., 2006). Afterwards, GDNF is restricted to specific muscular tissues such as the *cutaneus maximus* and* latissimus dorsi* in the forelimb as well as the *gluteus* and* iliopsoas* in the hindlimb. GDNF exerts specific effects on these MNs, since binding to its receptor induces the expression of the transcription factor PEA3, which governs soma positioning, target innervation and the formation of afferent synapses on the *cutaneous maximus* and* latissimus dorsi* (Haase et al., 2002; Vrieseling and Arber, 2006). Schwann cells also express GDNF at E12.5, but a function has not been identified (Gould et al., 2008). By E15, GDNF expression is regained throughout the body, but only in muscular intrafusal fibers that will be innervated by γ-MNs (Buss et al., 2006). In the lateral division of the lateral motor column (LMCl), which expresses high RET levels, GDNF also guides MN axons growing to the dorsal muscles via the interaction between RET and the immunoglobulin family member ISLR2 (Mandai et al., 2009). The changing and intermittent expression patterns of GDNF might help avoid “the candy-store effect” (i.e., the entrapment of elongating axons that remain in a location with a high GDNF concentration); axon entrapment prevents axons from crossing and/or reaching their final targets. For example, if GDNF is overexpressed by exogenous cells or released by a biodegradable material in a peripheral axonal injury model, axons sprout and grow towards the GDNF location; however, no farther growth is observed beyond that point, which impairs the recovery of these lesioned animals (Tannemaat et al., 2008; Hoyng et al., 2014).

The neurite sprouting action of GDNF in axonal injury models has been widely described, and it is the second most studied property of this trophic factor. New research has focused on using nanomaterials as scaffolds to deliver proper amounts of GDNF in the required time and space to promote recovery from sciatic nerve (Zhuang et al., 2016), recurrent laryngeal nerve (Hernandez-Morato et al., 2016; Wang et al., 2016) or dorsal root and spinal cord injury (Santos et al., 2016). Electrical stimulation of the tibial muscle after transection of the tibial nerve induces GDNF transcription, which is associated with reinnervation and functional recovery (Willand et al., 2016). Therefore, GDNF might have different roles, depending on the physiological and pathological scenarios.

The diverse actions of GDNF are so finely-tuned in space that the binding of GDNF to processes or soma elicits different responses. If the factor binds to neuronal soma, it promotes cell survival, but if GDNF interacts with a distal neurite, it facilitates axonal outgrowth (Zahavi et al., 2015). The mechanisms by which cells control signaling in such a meticulous way are remarkable. Studies investigating whether other trophic factors signal in similar fashion will be interesting.

Postnatally, GDNF is expressed at very low levels in the spinal cord; only a small group of cells known as Clarke’s column (or the posterior thoracic nucleus) located in lamina VII from T1 to L2-3 produce GDNF (Hantman and Jessell, 2010). Neurons in this column are involved in proprioception and their axons project via the spinocerebellar tract and receive information from primary SN, cortical neurons and GABAergic interneurons, but only the latter express GFRα1 receptor and are most probably the target of GDNF in this tissue (Hantman and Jessell, 2010). In early postnatal rats, GDNF is expressed at caudal levels in the ventrolateral position of the horn. The function is unclear but might be related to proprioception, similar to Clarke’s column (Ortega-de San Luis and Pascual, 2016). However, GDNF expression is eventually down-regulated, making this explanation most unlikely. These cells are detected as early as E11, and their real function might be associated with embryonic development of MNs. A similar case is observed at P0 within the pericenter of spinal cord; eventually, GDNF expression disappears in this region. The developmental function of this signal is to control midline-crossing axons (Charoy et al., 2012).

Myocytes, the target of MNs, also undergo changes related to GDNF exposure. This effect is highly noticeable at the point where both cells interact, namely, the neuromuscular junction (NMJ). In this case, the presence of GDNF in muscular cultures induces an accumulation of acetylcholine (ACh) receptors in the membrane, and if the muscle is co-cultured with MNs, this aggregation is concentrated in areas facing the NMJ (Figure 3). This accumulation of receptors is not due to *de novo* synthesis but to a rearrangement of already existing proteins via MAPK, Srk and cAMP signaling (Yang and Nelson, 2004). Although GDNF signaling is known to regulate several gene targets, *de novo* synthesis of ACh receptors was not detected in the latter study, possibly due to the short time period analyzed (8 h). Modifications in MN gene expression, such as genes in the ACh biosynthetic pathway, might require extra time. Studies examining whether the effects of GDNF observed on this binomial are also present in the postsynaptic moiety of other neurons responsive to this factor, such as neurons in the nigrostriatal pathway, would be equally interesting.

According to Buss, when GDNF is specifically overexpressed in muscle, an increase in the number of type IIa fibers (fast-twitch, fatigue-resistant) is observed; this phenomenon correlates with functional tests where myocytes derived from these mutants are fatigue-resistant. Despite the increase in the number of gamma neurons, the target intrafusal fibers were not modified. A possible explanation for unchanged number of intrafusal fibers is that the number of proprioceptive afferent neurons, which also contact intrafusal fibers to obtain a functional circuit (Figure 3), does not increase. Phenotypically, *MyoGdnf* animals possess impaired equilibrium and decreased total muscle strength and muscular mass (Buss et al., 2006).

The amyloid precursor protein (APP) was first described as the source of amyloidogenic peptides that play a central role in the pathophysiology of Alzheimer’s disease. APP has not been associated with a physiological role in the organism. As shown in a study by Stanga et al. (2016) using muscle cells, GDNF is modulated by APP, since depletion of this peptide diminished GDNF expression, precluding NMJ development by affecting muscular strength as well as neuron and myocyte differentiation. The remaining open questions are whether APP regulates GDNF expression in different types of neurons within the CNS and whether GDNF participates in Alzheimer’s disease.

## Enteric Nervous System

Neurons that control the peristaltic movement and secretion within the digestive tube (DT) arise from the neural crest. When these cells arrive in the gut, they are multipotent and express high levels of the GDNF co-receptor RET. Researchers isolated neural crest precursors and observed that these cells proliferate in the presence of GDNF when obtained at E12 (Chalazonitis et al., 1998; Taraviras et al., 1999), but not at E14 or E16; instead, at these later periods, GDNF inhibits glial differentiation and simultaneously induces the expression of TrkC, the main NT-3 receptor (Figure 3). However, the absence of NT-3 diminishes the final number of enteric neurons in the bowel (Chalazonitis et al., 1994). Nonetheless, this affliction is not as severe as in GDNF KO animals. Remarkably, earlier GDNF-sensitive precursors do not express GFRα1, but uses this receptor in *trans* (i.e., expressed by a nearby cell) to transmit GDNF signals. This *trans* activation of GDNF receptors is a well-known process involved in epithelial-mesenchymal interactions occurring during kidney induction, where GDNF has an important role (Chalazonitis et al., 1998). The absence of GDNF during development produces a deficiency in the enteric nervous system (ENS), a condition known as Hirschprung disease (Moore et al., 1996; Pichel et al., 1996; Sanchez et al., 1996; Treanor et al., 1996). This defect is probably not due to a lack of trophic support but might involve alterations in the expansion/induction of enteric neurons. In addition, RET is a dependent receptor; thus, two different signal outputs are observed, depending on whether the ligand is bound (Bordeaux et al., 2000). GDNF bound to RET induces proliferation, whereas RET without GDNF induces apoptosis. In any case, the absence of RET accounts for the highest proportion of cases of Hirschprung disease.

GDNF not only exerts effects during development but also on the adult gastrointestinal tract. This function seems to be associated with the immune response and inflammation, since the injured intestine expresses higher GDNF levels that promote the preservation of the epithelial barrier (Steinkamp et al., 2003). However, as shown in the study by Zeng et al. (2010) of an animal model of induced colitis, GDNF also has specific functions in the ENS, such as inhibiting the delayed-inward rectification potassium current and enhancing Htr3a expression. 5HT modulates peristaltic movements, the 5HT response is enhanced following GDNF treatment, resulting in higher peristaltic movements. In cultures of ENS from these experimental animals, GDNF also promotes synaptogenesis and neuronal communication (Zeng et al., 2010). Hence, GDNF might participate in pathological conditions such as chronic inflammatory bowel disease.

## Regulation of Pain and Nociception

Other cells sensitive to the non-survival effects of GDNF include neurons from the superior cervical ganglia (Coulpier et al., 2002) and dorsal root ganglia (DRG; Thang et al., 2000; Doxakis and Davies, 2005). For instance, when a nerve is transected and GDNF is applied, SN from the DRG increase their expression of NaN and SNS subunits of Na^+^ channels. These subunits constitute the tetrodotoxin-resistant Na^+^ channels, which are expressed at high levels in nociceptive neurons of the DRG. The expression of these channels avoids the decay of conduction velocity after axotomy (Cummins et al., 2000); hence, and as other authors have proposed, GDNF modulates nociception (Merighi, 2016; Salio and Ferrini, 2016).

Pain is present in many diseases as an unspecific symptom. In animal models, GDNF has been proven to control inflammatory and neuropathic pain, but effective methods of delivery are still under investigation. GDNF has been directly administered into the CNS, but adverse effects, such as migraine and the presence of antibodies directed against GDNF, have halted further investigation (Lang et al., 2006). GDNF-secreting cell grafts (Garbayo et al., 2013) and the nanoparticle-mediated delivery of the GDNF cDNA (Fletcher et al., 2011) have also been tested. Adenovirus and adeno-associated virus have been studied in clinical trials, but toxicity and inflammation were observed (Tarazi et al., 2014). Alternatively, the administration of gliafin, a peptide agonist for the GDNF receptor, induces RET phosphorylation, but studies related to pain reduction are still in progress (Garcia-Bennett et al., 2013). The small molecule XIB4035, which binds to GFRα1, has been topically administered to alleviate neuropathic pain in a rodent model of diabetes and achieved excellent results (Merighi, 2016). The mechanisms elicited by GDNF are not completely elucidated, but seem to involve a decrease in excitability and glutamate release (Salio et al., 2014), even under physiological conditions (Merighi, 2016). GDNF at least partially modulates pain by indirectly acting through a subpopulation of small- and medium-sized peptidergic neurons in the DRG that express calcitonin gene-related peptide and somatostatin. GDNF undergoes anterograde transport from these neurons to the dorsal horn of the spinal cord, increasing the pain threshold (Salio and Ferrini, 2016). Within the CNS, the *locus coeruleus* is the most important pain-modulator in the brain; GDNF modulates noradrenaline secretion from this region to the spinal cord and promotes analgesia in a chronic constriction injury model (Kimura et al., 2015). Thus GDNF modulates pain at different levels (Figure 3). Long-term potentiation modifies the pain threshold at the spinal cord level; this cellular change leads to hyperalgesia. High-frequency electrical stimulation induces long-term potentiation and molecular changes within the dorsal horn; for example, inflammatory molecules, such as IL-1β and iNOS, and other pain-regulating proteins, such as GDNF, are up-regulated (Pedersen et al., 2010). However, under these conditions, the effect of GDNF is difficult to understand, since researchers have not determined whether GDNF up-regulation favors or counteracts the installment of pain. GDNF has also been shown to enhance the capsaicin-stimulated release of calcitonin-related peptides, which produce thermal hyperalgesia (Schmutzler et al., 2009). The effects of GDNF on different types of pain must be studied further.

Delayed-onset of muscular soreness (DOMS) was thought to be caused by lactic acid accumulation, but this theory has been challenged. The current view includes microrupture of muscular fibrils (Cheung et al., 2003), and new mechanisms associated with inflammation have been discovered. Eccentric contraction induces Cox-2 activation and subsequent GDNF release from satellite cells, which are muscular stem cells. Released GDNF sensitizes Aδ sensory fibers, which are responsible for the discomfort. Cox-2 inhibition or the administration of anti-GDNF antibodies diminishes DOMS (Figure 3; Mizumura and Taguchi, 2016). The administration of anti-GDNF as a DOMS treatment is not practical, but Cox-2 inhibitors are widely used and highly effective (Forrest et al., 2015).

## Cancer and Other Pathological Conditions

Several types of neural crest-derived cells respond to GDNF during their ontogeny. Chromaffin cells from the adrenal medulla require TGFβ signaling, which in turns stimulates the expression of GDNF receptors. Chromaffin cells then differentiate and develop when GDNF is presented in* trans* (Förander et al., 2001). Kohno et al. (2011) have developed an immortalized chromaffin cell line from the adrenal medulla called tsAM5NE to study signaling molecules that induce neuronal differentiation. In these cells, GDNF induced the proliferation and acquisition of a neuronal-like phenotype (Figure 3). As mentioned above in this review, GDNF induces the expression of TrkC in enteric neurons, which are then able to respond to NT-3. Similar to the observations in the ENS, GDNF increases the expression of gp130, a LIF co-receptor, and the addition of this factor further promotes maturation (Kohno et al., 2011).

Neuroblastoma (NB) is a neural crest-derived tumor that usually arises from the adrenal gland (AG); it is composed of neurons and glia at different levels of differentiation, as well as a large pool of immature mitotic cells. These tumors are quite heterogeneous, ranging from spontaneous remission to unresponsive to any treatment (Behrman et al., 2000). One potential therapeutic approach is to induce the differentiation of NB. RET is present in some groups of these tumors and its activation induces differentiation, depending on whether they express low or high levels of RET. GDNF arrests NB cells in G0/G1 phase and together with CNTF, increases the expression of the NGF receptor TrkA. These three signals collaborate to: (i) decrease the expression of N-myc, thereby inhibiting proliferation; (ii) increase the proportion of G0 cells and decrease the number of cells in S phase; and (iii) further enhance the expression of neuron-specific proteins, such as SCG10, in HTLA 230 NB cells (Peterson and Bogenmann, 2004). Therefore, NB might be separated into a good or bad prognosis according to GDNF responsiveness and the capacity of this neurotrophic factor to diminish proliferation and induce differentiation. As shown in a similar study by Baldassarre, GDNF induces the expression of the CDK inhibitor p27^kip1^, halting proliferation in the embryonal carcinoma cell line NT2/D1 (Baldassarre et al., 2002); this effect contrasts the physiological role of GDNF in germ cells. Sertoli cells secrete GDNF and the protein reaches spermatagonial cells; GDNF induces the expression of Numb, an inductor of Notch degradation, thus preventing cells from differentiating and maintaining the stem cell pool (Braydich-Stolle et al., 2005). On the other hand, GDNF up-regulates the expression of Ki67, PCNA and cyclins in the rat C6 glioma cell line (Qu et al., 2015). What is the expression profile of cell cycle regulators among tumor cells with different responses to GDNF? The answer could provide insights into possible therapeutic targets.

The effects of GDNF on several neural functions or pathologies have been studied, although the role of this neurotrophin is not always clear. One example is attention-deficit/hyperactivity disorder, which is associated with high serum GDNF and NT-3 levels (Shim et al., 2015; Bilgiç et al., 2017). Researchers have not determined whether the high GDNF levels are the cause of the disorder or an effect.

Among the many hypothesis generated to explain the development of major depressive disorder, the neurotrophic hypothesis argues that an imbalance in trophic support primarily alters neurogenesis and subsequently generates depression. In this regard, the most substantial evidence for GDNF involvement in this pathology is the low peripheral GDNF levels (Lin and Tseng, 2015; Sharma et al., 2016). Nevertheless, alterations in the GDNF receptors and signaling pathways have been also reported. The micro-RNA miR-511 down-regulates the GFRα1a isoform, but not the GFRα1b isoform; this imbalance in the ratio of the two isoforms alters the behavior of basolateral amygdala neurons, inhibiting the expression of doublecortin, a marker of neuroblasts/early born neurons and a plastic phenotype (Maheu et al., 2015). Researchers do not completely understand how these alterations in GDNF signaling are associated with this disorder.

## Conclusions

GDNF was initially described as a molecule with a specific action: to promote the survival of a particular subset of cells, midbrain DANs. Since this first description, several neural and non-neural linages have proved to respond to GDNF; this growth factor has a variety of functions in addition to promoting survival, thus converting a neurotrophic molecule to pleiotropic factor. Therefore, GDNF has entered the group of molecules that participate in a great variety of cellular functions, such as TGFβ, interleukins, FGFs, etc. Thus, the function of GDNF can only be described in a specific context. In this review, we did not describe the effects of GDNF on epithelial-mesenchymal interactions in kidney formation, the ENS, and in craniofacial derivatives from the branchial arches. These functions highlight the importance of GDNF in the organogenesis of several systems in the organism.

Moreover, GDNF exerts effects such as differentiation, maturation and survival that conceptually are different but are actually difficult to separate, particularly during development. For instance, GDNF favors the expression of dopaminergic markers and survival-promoting proteins, such as Bcl-2, at the same time in the same space (Rolletschek et al., 2001; Lei et al., 2011).

On the other hand, we may always wonder which factor or cocktail of factors is better at producing more neurons in differentiation protocols; however, a more appropriate question might be which particular spatiotemporal combination is better for a given phenotype. This question has been partially studied in stem cell-derived neural differentiation protocols (Storch et al., 2001; Riaz et al., 2004; Sun et al., 2004; Wood et al., 2005; Zihlmann et al., 2005). During development, these factors are expressed in a specific spatiotemporal pattern, a very different condition from the addition of “differentiation cocktails”, where many factors are added simultaneously. Dissecting the effects of each trophic factor is part of the route to completely understand differentiation processes, such as the pleiotropic role of GDNF in ESC-derived MNs differentiation (Cortés et al., 2016).

GDNF signaling indisputably possesses potential therapeutic value for several pathologies, such as Parkinson’s disease or amyotrophic lateral sclerosis, among other diseases, where it has been tested using molecular and cellular approaches (Richardson et al., 2011; Thomsen et al., 2014; Rolan et al., 2015). In addition, the GDNF amino acid sequence has been engineered with modifications to improve its bio-distribution and half-life and to decrease its immunogenicity (Smith et al., 2015). The understanding of the entire range of properties of GDNF and other trophic molecules could provide opportunities to investigate their broad actions and accelerate and/or improve therapies based on these factors.

## Author Contributions

DC: conception and design, compiling and summarizing articles, figures elaboration, manuscript writing, final approval of the manuscript. OAC-M: axonal growth and regeneration data, final approval of the manuscript. MJC-M: elaboration of figures, final approval of manuscript. IV: financial support, design, correction of style, manuscript writing, final approval of the manuscript.

## Conflict of Interest Statement

The authors declare that the research was conducted in the absence of any commercial or financial relationships that could be construed as a potential conflict of interest.
